# 

**Published:** 1887-04-16

**Authors:** 


### The Hospital.

**An Institution, Family, and Parochial Journal of Hospitals, Asylums, and all Agencies for the Care of the Sick, Criticism and News.**

SATURDAY, APRIL 16TH, 1887.

### EASTER EGGS.

(*To be continued.*)

### QUESTIONS AND ANSWERS.

All questions should be addressed to Mr. Howard J. Collins, Secretary, The Hospitals Association, Norfolk House, Norfolk Street. Strand, W.C.

### HOMES FOR CONVALESCENTS WITH WOUNDS.

Nurse Nicholls, of 25, Nicholas Street, Chester, writes:—"Will the Editor kindly send a list of convalescent homes, which take in patients who have wounds, and cannot dress them themselves? She has a patient in Chester who wishes to go into one."

*** The Jaffray Suburban Hospital, Gravelly Hill, Birmingham, is the only institution of the kind where we know, for certain, that cases with open wounds are received. Probably a letter addressed to Mr. John Jaffray, J.P., Park Grove, Edgbaston, Birmingham, the founder of this chronic hospital, would secure admission to this patient as an act of grace, although the institution referred to is intended mainly, if not entirely, for cases from Birmingham and the neighbourhood.

### The Literary Prize.

A PRIZE of **Two Guineas** will be given every month for the best story or incident based upon hospital, or asylum, or student life, or any incident connected with the professions of medicine or nursing.

### Hospital Housekeeping.

FORMS and full particulars of the Quilter Prizes under this head (see page 355 for 19th Feb.) may be had on application to the Editor of THE HOSPITAL, Norfolk House, W.C. All competitive essays to be sent in by 30th April, 1887. Prizes, £10 10*s*. and £5 5*s*.

### The Children's Corner.

**Third Quarterly Prize Competition.**

Began 2nd Apr., 1887 ; ends 25th June, 1887.

PRIZES.

FOR MOST CORRECT ANSWERS TO PUZZLES.

1st .. .. Thirty Shillings | 3rd .. .. Ten Shillings
2nd .. .. Twenty „ | 4th .. .. Five „

FOR NEATEST ANSWERS TO PUZZLES.

Five Shillings.

FOR BEST LITTLE LETTERS.

Ten Shillings.

**Puzzles—Third Week.**

No. 10. CONUNDRUM. Which travels faster, heat or cold ; and why ?

No. 11. DECAPITATION.

My *whole* is what must ever be
Of use to those who go to sea ;
Cut off my head, and there behold
An animal just four years old ;
Cut off my tail, and then I'll be
A fish—not taken from the sea.

No. 12. FRENCH PUZZLE. What French word is that which has all five vowels and only one consonant ?

No. 13. PUZZLE SENTENCE. His illness lasted from A till e.

No. 14. DROPPED LETTER PUZZLE.

O×e t×××g at a t××e,
A×d t××t d××e w××l,
Is a v××y g××d r××e,
As m××y c×n t××l.

**Letters—Third Week.**

The Letter-subject for next week will be "How I spent Easter."

**Results of First Week—Puzzles.**

No. . DOUBLE MESOSTICH.

P

h U t d i Z z y

P U Z Z L E S

h i L l s t E n

S

No. 2. ENIGMA. Well-ling-ton=Wellington.

No. 3 FLOWER PUZZLE. Car-nation=Carnation.

No. 4. CONUNDRUM. A watch.

No. 5. MISSING VOWEL PUZZLE. "A still tongue makes a wise head."

*All right*—R. Epton, Peeler, Nordisa, Gom, A. C. K., Scrooge, Dot; *Four right*—Clytie, Alsie, Agatha, A. G. S. McCulloch, Tim, P.M., Umslopogaas, Isobel, Mikado, Ethel, Ellie, Skye.

**Letters.**

I am very pleased to welcome several new little friends, who have entered the lists, evidently intent on winning. One or two new competitors have not given me their full Christian names ; will they please do so next week ?

The letters on "Kindness to Animals" have pleased me so much, for they all of them evince a true love for God's dumb creatures—a love which I wish was that of every English boy and girl. This is a subject in which I am much interested, but, as I am anxious to print some of the letters I have received, I must not say more myself. Here is an interesting letter from "Bohemian Girl" :—

DEAR MR. EDITOR,—We always try to be kind to animals, for then they grow quite fond of us. We have a dog called Puck ; he has such funny ways. We have all made a great pet of him, especially my youngest brother. Frank always kisses Puck, and says good-night to him. In the summer my brothers used to play at soldiers in the garden, and when it was Frank's turn to be wounded and put on a stretcher to be carried to the hospital, Puck used to walk by the side of it. He will not allow anyone to hit Frank, not even papa.

Yours truly, BOHEMIAN GIRL, aged 13

12, Upper Tichborne Street, Leicester.

A new competitor, who has chosen an extraordinary *nom de plume*, writes me :—

DEAR MR. EDITOR,—I think you will say that we must have learnt to be kind to animals here, as a friend has asked us to take care of two dogs which are very great pets. One of them is a Fox Terrier, and the other a Blenheim Spaniel, and, of course, we have to take very great care of them. Then we have a dog and cat of our own, which are very fond of us, and my little brother's two doves feed from our hands and allow us to carry them about without the slightest fear. We certainly have a little trouble in making the animals kind to each other, as the dogs often quarrel over their biscuits, and the cat shows a desire to eat up the doves ; but I think, with a little patience, we shall soon get them to live together as a very happy family.

I am, dear Mr. Editor, yours truly, UMSLOPOGAAS, aged 12.

25, Dornton Road, Balham.

Peeler always writes thoughtfully, and his letters invariably display great accuracy of observation. Here is one just to hand :—

DEAR MR. EDITOR,—It is a pleasing thing to see a London cabman caring for his horse ; and to see the horse caring for the master. The horse works a great deal more by kindness than by brutality. Take, for instance, the Arab : his horse is treated like one of his family, and the result is that he is docile and faithful. Many are the instances of fidelity in animals, but I think the most touching to be found is the story of "Androcles and the lion" ; how well that lion knew "one good turn deserves another." How astonished those Romans must have been to see the animal, who was roaring for his prey but a few minutes ago, now fawning on the man who had shown kindness to him in his pain.

He prayeth best, who loveth best
All things both great and small ;
For the dear God who loveth us,
He made and loveth all.

Yours faithfully, PEELER, aged 18.

Eaton, Chester.

One mark each :—Halford, Isobel, Peeler, Mikado, Umslopogaas, Bohemian Girl, Ethel, Ellie, Scrooge, Skye.

**Rules.**

Only one side of the paper should be written upon.

The address and full Christian name and surname, and the age of the competitor, must be stated in all replies. A *nom de plume* may be added for the purposes of publication.

The first prize may not be taken by the same competitor more than once in any one year. In the event of the winner of the first prize again scoring highest, the second prize will be awarded, the next competitor receiving the first.

Answers must be addressed to the "PUZZLE EDITOR," THE HOSPITAL, Norfolk House, Norfolk Street, Strand, W.C., not later than Thursday next. The "Children's Corner" Coupon (see advertisement pages) must be enclosed.

### Amusements with Profit.

### PRIZE COMPETITIONS.

**No. 25. Double Acrostic.**

A *tyrant*, who, deprived of crown, to teaching took ;
A kind of brass, known also as *Mosaic gold ;*
A creature—*half human, half equine* in its look ;
One of a *sect* who *murder* as a rite did hold ;
'Tis fam'd *Kosala's site*, they say, in distant Ind ;
To land, a *Red Rose*, then a *White*, sought out this nook ;
Close to the *Strand* my *precincts* you will doubtless find.

(The initials and finals give the title of a place where surgical operations are performed or where medicines are prepared.)

**No. 26. Mathematical Question.**

If one and a half cats kill one and a half mice in one and a half minutes, how many cats will kill one hundred mice in fifty minutes ?

**Answers.**

No. 21. DOUBLE ACROSTIC.

| | | |
|---|---|---|
| M | acduf | F |
| E | xcalibu | R |
| T | ouchston | E |
| R | osetta Ston | E |
| O | mag | H |
| P | izarr | O |
| O | siri | S |
| L | am | P |
| I | lliman | I |
| T | albo | T |
| A | rmad | A |
| N | euchate | L |

Correct answers have been received from Perseverance, Gip, Ostrich, Gretta, Boss, Ellice, Peeler, Mater, K. M., May, A. M. E.

No. 22. MEDICINE, ITS USE AND ABUSE.

The majority of our competitors have given instructive and very interesting answers.

Best answers have been received from Gretta, Ellice, Peeler, Perseverance, Mater, K. M., Ostrich, Gip, A. M. E.

Answers must be addressed to THE PRIZE EDITOR, AMUSEMENTS WITH PROFIT, THE HOSPITAL, Norfolk House, Norfolk Street, Strand, W.C., not later than the *first* post on Saturday next. The full name and address must in all cases be given, but a *nom de plume* may be added for publication. Only one side of the paper should be written on. The "Amusements with Profit" Coupon (see advertisement pages) must be enclosed with replies

⁂ Next week will commence our Second Quarterly Prize Competition.

### THE IN- AND OUT-PATIENTS' HOSPITAL DIARY.

This Diary is so arranged as to make some portion of it appear every week, and the whole in each monthly part. It has been very difficult to compile, and corrections will at all times be gratefully received. It has been suggested that similar information about the larger Provincial and Scotch Hospitals would be useful to many readers. We should be glad to hear if this is felt to be the case.

### CHILDREN'S DISEASES.—*continued.*

| HOSPITALS AND TERMS OF ADMISSION. | PHYSICIANS, SURGEONS, AND MEDICAL OFFICERS, WITH DAYS AND HOURS OF ATTENDANCE. |
|---|---|
| SAMARITAN FREE HOSPITAL FOR WOMEN AND CHILDREN, Lower Seymour Street, W.; Branch, 1, Dorset St., W. Sec., Mr. G. Scudamore. In-patients must be between 2 and 12. Out-patients Department (free) is at Edward's Mews, Duke Street, W. | *In-Physician*, Dr. W. H. Day. Daily, 2. *In-Surgeon*, Mr. W. A. Meredith. Daily, 2. *Out-Physicians*, Drs. M. Prickett and Amand Routh, W. S. 1.30. *Out-Surgeons*, Messrs. W. A. Meredith and J. D. Malcolm, M. Th.; A. H. G. Doran and W. S. A. Griffith, Tu. F., 1.30 |
| VICTORIA HOSP. FOR CHILDREN, Queen's Road, Chelsea, S.W. Sec., Capt. W. C. Blount, R.N. Age of boys, 2 to 12 years; age of girls, 2 to 16. Admission for in-patients by subscriber's letter. Accidents and urgent cases free at all times | *In-Physicians*, Drs. J. Evans, M. Th. 2; Ridge-Jones, Tu. F. 2. *In-Surgeons*, Messrs. Pick, Tu. F. 9.15; Clutton, M. Th. 4.30. *Out-Physicians*, Drs. A. Venn, W. S. 9.30; C. Fox, M. Th. 1.30; F. D. Drewitt, Tu. F. 9; J. Philpot, M. Th. 9. *Out-Surgeons*, Messrs. F. Churchill, Tu. F. 10; W. Pye, M. Th. 9.30 |
| WEST LONDON HOSPITAL, Hammersmith rd, W. | Daily, 2. *See* General Hospitals |

### CONSUMPTION AND DISEASES OF THE CHEST.

| HOSPITALS AND TERMS OF ADMISSION. | PHYSICIANS, SURGEONS, AND MEDICAL OFFICERS, WITH DAYS AND HOURS OF ATTENDANCE. |
|---|---|
| CITY OF LONDON HOSPITAL FOR DISEASES OF THE CHEST, Victoria Park, E. Sec., Mr. T. Storrar Smith. By subscriber's letter available for 6 weeks. | *In-Physicians*, Drs. J. Thorowgood, S.; E. Smith, M.; J. Fothergill, W.; S. West, F.; G. A. Heron, W.; V. D. Harris, Tu. Hour, 2. *Out-Physicians*, Drs. V. D. Harris, Tu.; J. A. Ormerod, Th.; E. C. Beale, Tu. F.; B. Fenwick, W. S.; A. Money, W. S.; L. E. Shaw, M. Th. Hour, 2 |
| HOSPITAL FOR CONSUMPTION, Fulham road, Brompton, S.W. Sec., Mr. H. Dobbin. Admission by letter of recommendation, but, where unobtainable, application may be made to the Secretary at the Hospital. | *In-Physicians*, Drs. E. S. Thompson, M. Th.; C. T. Williams, M. Th.; R. D. Powell, Tu. F.; J. Tatham, W. S.; R. E. Thompson, W. S.; F. T. Roberts, Tu. F. Hours, 2 to 4. *In-Surgeon*, Mr. R. J. Godlee, F. *Out-Physicians*, Drs. T. H. Green, J. M. Bruce, M. Th.; J. K. Fowler, W. S.; P. Kidd and C. Y. Biss, Tu. F.; T. D. Acland, W. S. Hour, 12 |
| NORTH LONDON HOSPITAL FOR CONSUMPTION AND DISEASES OF THE CHEST, Mount Vernon, Hampstead, N. Sec., Mr. L. F. Hill. Admission by subscriber's letter available for 6 weeks. | *In-Physicians*, Drs. A. Evershed, Tu.; B. O'Connor, F.; D. Pidcock, F. Hour, 10.30. *Out-Physicians*, Drs. B. O'Connor, D. Pidcock, J. E. Squire. Daily, 2 (attend at Out-patients' Branch, 216, Tottenham Court Road, W.) |
| ROYAL HOSPITAL FOR DISEASES OF THE CHEST, 231, City Rd., E.C. Sec., Mr. John J. Austin. By Governor's letter, available for six weeks | *In-Physicians*, Drs. Hensley, W.; Gilbart-Smith, M.; Finlay, Th. *In-Surgeon*, Mr. A. Pearce-Gould, M. Tu. W. Th. F. 2, S. 9. *Out-Physicians*, Drs. White, Tu. F.; Pringle, M. Th.; O. Browne, W. S.; A. Davies, M. F.; H. Habershon, W. S.; E. Stewart, Tu. Th. Hour, 1; S. 9 |
| ROYAL NATIONAL HOSP. FOR CONSUMPTION AND DISEASES OF THE CHEST, Ventnor. Offices, 34, Craven St., Strand, W.C. Sec., Mr. E. Morgan. Admits only incipient cases by Governor's letter. | *In-and Out-Physicians*, Drs. J. G. Sinclair Coghill, and R. Robertson attend daily (at Ventnor) on out-patients at 11; on in-patients at noon. *In- and Out-Surgeons*, Messrs. Whitehead, Williamson |

### EAR CASES.

| HOSPITALS AND TERMS OF ADMISSION. | PHYSICIANS, SURGEONS, AND MEDICAL OFFICERS, WITH DAYS AND HOURS OF ATTENDANCE. |
|---|---|
| CENTRAL LONDON THROAT AND EAR HOSP., Gray's Inn Rd., W.C. Sec., Mr. R. Kershaw. Free, but when able, a small payment is expected | *In- and Out-Surgeons*, Mr. Lennox Browne, M, Th. 2.30; Dr. Orwin, W. S. 2.30; Dr. Grant, Tu. 5, Th. 2.30; Messrs. P. S. Jakins, M. W. 2.30; F. 5; T. Carmalt Jones, M. Th. S. 2.30 |
| CHARING CROSS HOSPITAL, West Strand, W.C. | *In- and Out-Surgeon*, Mr. Cantlie, F. 9 |
| GREAT NORTHERN CENTRAL HOSPITAL, Caledonian Rd, N. | *In- and Out-Surgeon*, Mr. A. E. Cumberbatch, W. 9 |
| GUY'S HOSPITAL, St. Thomas's Street, S.E. | *In- and Out-Surgeon*, Mr. Purves, Tu. F. 12.30 |
| HOSPITAL FOR DISEASES OF THE THROAT, Golden Square, W. Sec., Mr. W. T. Sharp | *Physicians*, Drs. Wolfenden, Tu. F. 2.30; Macdonald, Tu. F. 6.30; Bond, W. S. 2.30. *Surgeon*, Mr. T. M. Hovell, M. Th. 2.30 |

### EAR CASES—*Continued.*

| HOSPITALS AND TERMS OF ADMISSION. | PHYSICIANS, SURGEONS, AND MEDICAL OFFICERS, WITH DAYS AND HOURS OF ATTENDANCE. |
|---|---|
| KING'S COLLEGE HOSPITAL, Portugal Street, W.C. | *In- and Out- Surgeon*, Mr. Pritchard Th. 2.30 |
| LONDON HOSPITAL, Whitechapel Road, E. | *In- and Out-Surgeons*, Dr. Woakes, Mr. T. M. Hovell, S. 9 |
| MIDDLESEX HOSPITAL, Berners Street, W. Free. | *Out-Surgeon*, Mr. Hensman, Tu. 9 |
| NATIONAL HOSP. FOR THE PARALYSED, ETC., Queen Sq., W.C. | *In- and Out-Surgeon*, Mr. A. E. Cumberbatch, W. 2 |
| NORTH WEST LONDON HOSPITAL, 18, Kentish Town Rd. | *Out-Surgeon*, Mr. Collier, M. 1.30 |
| ST. BARTHOLOMEW'S HOSPITAL, Smithfield, E.C. | *Surgeon*, Mr. Cumberbatch, Tu. F. 2 |
| ST. GEORGE'S HOSPITAL, Hyde Park Corner, S.W. | *In- and Out-Surgeon*, Sir W. B. Dalby, Tu. 1.30 |
| ST. MARY'S HOSPITAL, Cambridge Place, Paddington, W. | *In- and Out-Surgeon*, Mr. G. P. Field, W. S. 9.30 |
| ST. THOMAS'S HOSPITAL, Westminster Bridge Road, S.E. | *Out-Surgeon*, Mr. Clutton, M. 1.30 |
| UNIVERSITY COLLEGE HOSPITAL, Gower Street, W.C. | *In- and Out-Surgeon*, Mr. Barker, Th. 9 |
| WESTMINSTER HOSPITAL, Broad Sanctuary, S.W. | *In- and Out-Surgeon*, Mr. Barrow, Tu. F. 9 |

### EYE DISEASES.

| HOSPITALS AND TERMS OF ADMISSION. | PHYSICIANS, SURGEONS, AND MEDICAL OFFICERS, WITH DAYS AND HOURS OF ATTENDANCE. |
|---|---|
| CENTRAL LONDON OPHTHALMIC HOSP., 238A, Gray's Inn Rd., W.C. Sec., Mr. G. H. Leah. Admission free | *Surgeons*, Messrs. T. Archer, G. Abbott, W. Charnley, W. Jessop, E. Clarke, J. R. Kemp and Granville Barker (House Surgeon). Daily, 1 |
| EVELINA HOSP. FOR SICK CHILDREN, Southwark Bridge Rd. | *Surgeon*, Dr. W. Brailey, Th. 1 |
| GREAT NORTHERN CENTRAL HOSPITAL, Caledonian Rd., N. | *Surgeon*, Mr. Hutchinson, Jnr., Tu. F. 9.30 |
| GUY'S HOSPITAL, St. Thomas's Street, S.E. | *Surgeons*, Mr. Higgens, Dr. Brailey, Tu. F. 12.30 |
| HOSPITAL FOR SICK CHILDREN, 49, Gt. Ormond Street, W.C. | *Surgeon*, Mr. R. Marcus Gunn, Tu. 2 |
| KING'S COLLEGE HOSPITAL, Portugal Street, W.C. | *Surgeon*, Mr. McHardy, M. W. Th. 1.30 |
| LONDON HOSPITAL, Whitechapel Road, E. | *Surgeons*, Messrs. Waren Tay, S. 9; Eve, Tu. 9 |
| MIDDLESEX HOSPITAL, Berners Street, W. | *Surgeon*, Mr. Lang, Tu. F. 9 |
| NATIONAL HOSP. FOR THE PARALYSED, ETC. Queen Sq., W.C. | *Surgeons*, Messrs. R. Brudenell Carter, F. 4; R. Marcus Gunn, Tu. 4 |
| NORTH-WEST LONDON HOSP. 18, Kentish Town Rd., N.W. | *Surgeon*, Dr. W. Collins, F. 3.30 |
| PADDINGTON GREEN CHILDREN'S HOSP., Paddington Grn. | *Surgeon*, Mr. W. H. H. Jessop, Th. 9 |
| ROYAL FREE HOSPITAL, Gray's Inn Road, W.C. | *Surgeon*, Mr. Mackinlay, M. F. 9 |
| ROYAL LONDON OPHTHALMIC HOSPITAL, Blomfield Street, Moorfields, E.C. Sec., Mr. R. J. Newstead. Admission free to the poor. | *Surgeons*, Messrs. J. W. Hulke, W. S.; G. Lawson, Tu. F.; J. Couper, Tu. F.; W. Tay, M. Th.; J. Tweedy, Tu. F.; E. Nettleship, W. S.; R. M. Gunn, W. S.; Lang, M. Th. Hours, 8 to 10 |
| ROYAL SOUTH LONDON OPHTHALMIC HOSP., 6, St. George's Circus, S.E. Sec., Mr. C. Comyn. Free to the necessitous poor; or by payment | *Surgeons*, Messrs. Watson, McHardy. Daily, 2 |
| ROYAL WESTMINSTER OPHTHALMIC HOSPITAL, King William Street, W.C. Sec., Mr. T. Beattie-Campbell. Poor and accidents free | *In- and Out-Surgeons*, Messrs. Power, M. F.; Frost, M. Th.; Rouse, Tu. S.; Hartridge, Tu. F.; Macnamara, M. Th.; Cowell, W. S.; Juler, W. S. Hour, 1 |
| ST. BARTHOLOMEW'S HOSPITAL, Smithfield, E.C. | *Surgeons*, Messrs. Power, Th. 2; Vernon, Tu. S. 2.30 |
| ST. GEORGE'S HOSPITAL, Hyde Park Corner, S.W. | *Surgeons*, Messrs. B. Carter, Frost, M. Th. 2; F. 1.15; W. S. 2 |
| ST. MARY'S HOSPITAL, Cambridge Place, Paddington. W. | *Surgeons*, Messrs. A. Critchett, H. Juler, Tu. F. 9.30 |
| ST. THOMAS'S HOSPITAL, Westminster Bridge Road, S.E. | *Surgeons*, Messrs. Nettleship, Tu. Th. F. 1.30; Lawford, M. W. 1.30 |
| UNIVERSITY COLLEGE HOSPITAL, Gower Street, W.C. | *Surgeon*, Mr. Tweedy, M. Tu. Th. F. 2 |
| WEST LONDON HOSPITAL, Hammersmith Road, W. | *Surgeons*, Messrs. B. Vernon, H. Dunn, Tu. Th. S. 2 |
| WESTMINSTER HOSPITAL, Broad Sanctuary, S.W. | *Surgeon*, Mr. Cowell, M. Th. 2 |
| WESTERN OPHTHALMIC HOSP. 153 and 155, Marylebone Rd., W. Sec., Mr. G. E. Manton. By letter or small payments; free to poor | *Surgeons*, Drs. Miller, M.; Collins, F.; Wheatly, M. Th.; Messrs. Carmal, Jones, W.; Buxton, Tu. F.; Nunn, W. S. Hour, 2 |

### THE IN- AND OUT-PATIENTS' HOSPITAL DIARY.

### ORTHOPÆDIC CASES.

| Hospitals and Terms of Admission. | Physicians, Surgeons, and Medical Officers, with Days and Hours of Attendance. |
|---|---|
| City Orthopædic Hospital, 27, Hatton Garden, E.C. Sec., Mr. E. Dereuth<br>Admission free | *Surgeons*, Mr. E. J. Chance and House-Surgeon. M. Tu. Th. F. 2.30. New cases Tu. F. 2.30 |
| National Orthopædic Hospital for the Deformed, 234, Gt. Portland St., W. Sec., Mr. H. Canning<br>Free to poor; others by letter or payment | *In-Physician*, Dr. C. Beevor<br>*In- and Out-Surgeons*, Messrs. F. R. Fisher, M. Th. 2; W. J. Roeckel, Tu. F. 2; E. M. Little, W. 1 |
| Royal Orthopædic Hospital, 297, Oxford Street, W. Sec., Mr. B. Maskell<br>By Governor's letter | *Surgeons*, Messrs. B. E. Brodhurst, H. A. Reeves, C. Read, W. E. Balkwill, H. F. Baker. Daily, 1 |
| St. Bartholomew's Hospital, Smithfield, E.C. | *Surgeon*, Mr. Walsham, M. 2.30 |
| St. George's Hospital, Hyde Park Corner, S.W. | W. 2. *See* General Hospitals |

### PARALYSIS, EPILEPSY, AND DISEASES OF THE NERVOUS SYSTEM GENERALLY.

| | |
|---|---|
| Hospital for Epilepsy and Paralysis, and other Diseases of the Nervous System, 32, Portland Terrace, Regent's Park Road, N.W. Sec., Mr. Howgrave Graham<br>By contributor's letter, or payment according to means, but free to necessitous poor | *In-Physicians*, Drs. J. Althaus, Th.; Hughes Bennett, M.; G. Ogilvie, Tu. Hour 2<br>*Out-Physicians*, Drs. Laycock, F.; W. H. Syers, W.; J. Althaus, Th.; G. Ogilvie, Tu.; H. Bennett, M. Hour, 2<br>*In- and Out-Surgeons*, Messrs. J. Bloxam, A. P. Gould, when required |
| National Hospital for the Paralysed and Epileptic, Queen Square, Bloomsbury, W.C. Sec. and Director, Mr. B. B. Rawlings<br>Most of the beds are reserved free to the necessitous poor; the others are for patients who contribute a Guinea a week | *In-Physicians*, Drs. J. S. Ramskill, Tu.; C. B. Radcliffe, M.; J. H. Jackson, F.; T. Buzzard, W. Hour, 2.30<br>*In-Surgeons*, Messrs. W. Adams, M. 3; V. Horsley, F. 2.30<br>*Out-Physicians*, Drs. T. Buzzard, W., H. C. Bastian, Tu.; W. R. Gowers. M.; D. Ferrier, F. 2.30; J. A. Ormerod, Tu. W.; C. E. Beevor, M; F. Hour, 2.30 |
| National Hospital for Diseases of the Heart and Paralysis, 32, Soho Sq., W. Sec., Capt. F. Handley<br>In-patients require recommendation of Life Governor. | *In-Physicians*, Drs. A. Duncan, J. Murray, Stretch Dowse, M. W. Th.<br>*In-Surgeon*, Mr. Graham Bennett, Th.<br>*Out-Physicians*, Drs. R. Verley, J. Murray, Stretch Dowse, Tu. Th. F. 3; M. W. 3 and 8<br>*Out-Surgeon*, Mr. G. Bennett, Th. 3 |
| West End Hospital for Diseases of the Nervous System, 73, Welbeck St., W. Hon. Sec., Mr. H A. Dowell<br>Children only as in-patients. Out-patients by letter or payment. | *In- and Out-Physicians*, Drs. H. Tibbits, M. Th. 1.30; Stretch-Dowse, Tu. W. 1.30; S. H. Armitage, W. Tu. F. 6<br>*In- and Out-Surgeon*, Mr. Benson |

### DISEASES OF THE SKIN.

| | |
|---|---|
| Charing Cross Hospital, West Strand, W.C. | *Physician*, Dr. Sangster, M. 2 |
| Guy's Hospital, St. Thomas Street, S.E. | *Physicians*, Drs. Pye-Smith, Hale-White, Tu. 12 |
| Hospital for Diseases of the Skin, 52, Stamford St., S.E. Sec., Mr. S. Hayman<br>Free to the necessitous; also by payment | *Physician*, Dr. F. J. Payne<br>*Surgeons*, Messrs. J. Hutchinson, Waren Tay, and Wyndham Cottle. (Out-patients seen M. W. 3 Tu. Th. F. 2) |
| King's College Hospital, Portugal Street, W.C. | *Physician*, Dr. Duffin, F. 1 |
| London Hospital, Whitechapel Road, E. | *Physician*, Dr. S. Mackenzie, Th. 9 |
| Middlesex Hospital, Berners Street, W. | *Physician*, Dr. R. Liveing, Tu. 4 |
| North-West London Hosp. 18, Kentish Town Rd., N.W. | *Physician*, Dr. J. Stowers, Tu. 1.30 |
| Paddington Green Children's Hospital, Paddington | *Physician*, Dr. T. Colcott Fox, S. 9.15 |
| St Bartholomew's Hospital, Smithfield, E.C. | *Surgeon*, Mr. H. Cripps, F. 1.30 |

### DISEASES OF THE SKIN.—*Continued.*

| Hospitals and Terms of Admission. | Physicians, Surgeons, and Medical Officers, with Days and Hours of Attendance. |
|---|---|
| St. George's Hospital. Hyde Park Corner, S.W. | *Physician*, Dr. Cavafy, W. 1.30 |
| St. John's Hospital for Diseases of the Skin, 45, Leicester Sq., W.C. Branch, Markham Square, Chelsea, S.W. Sec., Mr. St. Vincent Mercier. In-patients by letter; out-patients free or by small payment | *Physicians*, Drs. Robinson, Dow, Harries, Campbell, Bourns. Daily 2 and 7. Markham Sq. Branch, M. 10 and Th. 10 and 7<br>*Surgeon*, Mr. J. Milton, Tu. |
| St. Mary's Hospital, Cambridge Place, Paddington, W. | *Surgeon*, Mr. Morris, M. Th. 9.30 |
| St. Thomas's Hospital, Westminster Bridge Road, S.E. | *Physician*, Dr. Payne, W. 1.30 |
| University College Hospital, Gower Street, W.C. | *Physician*, Dr. Crocker, W. 1.30, S. 9.15 |
| Westminster Hospital, Broad Sanctuary, S.W. | *Physician*, Dr. T. C. Fox, W. 1.30 |

### STONE AND OTHER DISEASES OF URINARY ORGANS.

| | |
|---|---|
| London Hospital, Whitechapel Road, E. | Daily, 1 30. *See* General Hospitals |
| St. Peter's Hospital for Stone and Urinary Diseases, Henrietta Street, W.C. Sec., Mr. W. E. Scott.<br>Admission free. Only women and children are seen on Fridays. Operation day, Wednesday | *In-Surgeons*, Messrs. W. J. Coulson, F. R. Heycock.<br>*Out-Surgeons*, Messrs. E. H. Fenwick, M. 2, S. 4 to 7; F. S. Edwards, M. 5 to 7, Th. 2; Heycock, Tu. 2, W. 5 to 7, F. 2 |

### DISEASES AND IRREGULARITIES OF THE TEETH.

| | |
|---|---|
| Belgrave Hosp. for Children, 77 & 79 Gloucester St., Pimlico | *Surgeon*, Mr. G. W. Payne, W. 9 |
| Charing Cross Hospital, West Strand, W C. | *Surgeon*, Mr. Fairbank, M. W. F. 9.0 |
| Dental Hospital of London, Leicester Sq., W.C. Sec., Mr. J. F. Pink<br>Poor free. For special operation a Governor's letter is required | *Surgeons*, Messrs. Canton, Gregson, Hepburn, S. Bennett, Underwood, Woodhouse, Hern, Matheson, Parkinson, Read, Rogers, Truman. Daily, 9 to 11 |
| Evelina Hosp. for Sick Children, Southwark Brdg. Rd. | *Surgeon*, Mr. R. Denison Pedley, Tu. 9 |
| German Hospital, Dalston lane, Dalston, E. | *Surgeons*, Messrs. A. P. Reboul, C. West, Th. 10 to 11 |
| Great Northern Central Hospital, Caledonian Rd, N. | *Surgeon*, Mr. E. Keen, W. 1.30 |
| Guy's Hospital, St. Thomas Street, S.E. | *Surgeons*, Messrs. Moon, Pedley, Tu. Th. F. 12.30 |
| Hospital for Consumption, Brompton, S.W. | *Surgeon*, Mr. C. J. Noble, W., and when wanted |
| Hospital for Sick Children, Gt. Ormond Street, W.C. | *Surgeon*, Mr. Cartwright, W. 9 |
| King's College Hospital, Portugal Street, W.C. | *Surgeon*, Mr. Cartwright, Tu. F. 10 |
| London Hospital, Whitechapel Road, E. | *Surgeon*, Mr. A. W. Barrett, Tu. 9 |
| London Temperance Hosp., Hampstead Road, N.W. | *Surgeon*, Mr. Adolphus Alexander, M. 2 |
| Metropolitan Free Hospital, Gray's Inn Road, W.C. | *Surgeon*, Mr. G. Curnock, Tu. Th. S. 9 |
| Middlesex Hospital, Berners Street, W. | *Surgeons*, Messrs. Bennett, M. 9.30; W. 9; Hern, W. 9, F. 9.30 |
| National Dental Hospital, 149, Gt. Portland Street, W. Sec., Mr. A. G. Klugh.<br>The poor, and all urgent cases admitted free; others by Subscriber's letter | *Surgeons*, Messrs. H. Weiss, W. Weiss, M.; A. Smith, G. Bradshaw, Tu.; G. A. Williams, M. Davis, W.; A. F. Canton, H. G. Read, Th.; T. Gaddes, W. S. Thomson, F.; H. Rose, W. R. Humby, S. Hours 9 to 11 |
| National Orthopædic Hospital, 234, Gt. Portland st, W. | *Surgeon*, Mr. H. Harris |

